# Prevalence and Risk Factors of Augmented Renal Clearance: A Systematic Review and Meta-Analysis

**DOI:** 10.3390/pharmaceutics14020445

**Published:** 2022-02-19

**Authors:** Fatma Hefny, Anna Stuart, Janice Y. Kung, Sherif Hanafy Mahmoud

**Affiliations:** 1 Faculty of Pharmacy and Pharmaceutical Sciences, University of Alberta, Edmonton, AB T6G 2E1, Canada; hefny@ualberta.ca (F.H.); astuart@ualberta.ca (A.S.); 2 John W. Scott Health Sciences Library, University of Alberta, Edmonton, AB T6G 2R7, Canada; janice.kung@ualberta.ca

**Keywords:** augmented renal clearance, critically ill, glomerular hyperfiltration, neurocritical care, GFR

## Abstract

Kidney function assessment in the critically ill overlooks the possibility for hyperfunctioning kidneys, known as augmented renal clearance (ARC), which could contribute to therapeutic failures in the intensive care unit (ICU). The aim of this research is to conduct a systematic review and meta-analysis of prevalence and risk factors of ARC in the critically ill. MEDLINE, Embase, Cochrane Library, CINAHL, Scopus, ProQuest Dissertations and Theses Global databases were searched on 27 October 2020. We included studies conducted in critically ill adults who reported the prevalence and/or risk factors of ARC. We evaluated study quality using the Joanna Briggs Institute appraisal tool. Case reports, reviews, editorials and commentaries were excluded. We generated a random-effects meta-analytic model using the inverse variance method and visualized the pooled estimates using forest plots. Seventy studies were included. The pooled prevalence (95% CI) was 39% (34.9–43.3). Prevalence for neuro, trauma, mixed and sepsis ICUs were 74 (55–87), 58 (48–67), 36 (31–41) and 33 (21–48), respectively. Age, male sex and trauma were associated with ARC with pooled OR (95% CI) of 0.95 (0.93–0.96), 2.36 (1.28–4.36), 2.60 (1.21–5.58), respectively. Limitations included variations in ARC definition, inclusion and exclusion criteria and studies design. In conclusion, ARC is prevalent in critically ill patients, especially those in the neurocritical care and trauma ICU population. Young age, male sex and trauma are risk factors for ARC in those with apparently normal renal function. Further research on optimal dosing of drugs in the setting of ARC is warranted. (Prospero registration: CRD42021246417).

## 1. Introduction

Critical illness is unique for its complex nature, which very often requires a range of professional expertise to provide the most comprehensive care possible, hence the need for a multidisciplinary approach. When assessing a patient’s kidney function, particularly in a critical care setting, clinicians typically consider one of two possibilities: either normal renal function, or renal impairment, with most of the attention paid towards dosing adjustments in the presence of impaired renal function and/or the use of renal replacement therapy. This conventional view might in fact be overlooking a third category of patients who may exhibit hyperfunctioning kidneys or what is known as augmented renal clearance (ARC). This phenomenon, while not yet fully understood, may potentially be the rationale behind a range of therapeutic failures for renally-eliminated drugs [1,2,3]. This is mainly due to the fact that ARC is typically undetected unless clinicians proactively monitor for its presence and the lack of solid evidence on the dosing of renally-eliminated medications subject to an accelerated elimination, leading to subtherapeutic levels and sub-optimal outcomes. The pathophysiology of ARC is largely unknown, but it is thought to be closely tied to the vigorous sympathetic response associated with severe critical illness, alterations in vascular tone, cardiac output and major organs blood flow, resulting in a hyperdynamic state and augmented glomerular filtration rate [4,5]. This is in addition to the effects of administration of fluids and vasopressors aimed at maintaining organ perfusion [5,6]. ARC has most commonly been defined as a creatinine clearance (CrCl) higher than 130 mL/min/1.73 m^2^ [7,8,9]. However, there is not yet an agreed-upon cut-off for the CrCl above which a patient is diagnosed with ARC, nor a staging system for patients exhibiting CrCl more than 150 mL/min/1.73 m^2^ or even 200 mL/min/1.73 m^2^, analogous to renal impairment stages.

In recent years, there has been a growing number of reports recognizing the significance of ARC [4,10]. ARC prevalence has been reported to range from 18 to 80% in the general critically ill population [4,11,12,13,14,15,16,17,18]. However, reported studies varied in their patient population, sample sizes, inclusion and exclusion criteria and ARC definition, thus, impeding accurate identification of ARC prevalence and risk factors among intensive care unit (ICU) patients. Therefore, the aim of this research is to conduct a systematic review and meta-analysis of the available literature on ARC and to attempt to provide pooled estimates of its prevalence and contributing risk factors in various critically ill populations. To our knowledge, this is the first combined systematic review and meta-analysis of ARC in the critically ill. Our work represents a step towards defining the prevalence and risk factors of ARC, facilitating early identification of those at risk for ARC allowing timely medication optimization.

## 2. Materials and Methods

This review was conducted following the Preferred Reporting Items for Systematic Reviews and Meta-Analyses (PRISMA) checklist [19]. This review was registered in international prospective register of systematic reviews (PROSPERO). Registration number CRD42021246417 and protocol can be accessed in the following link: https://www.crd.york.ac.uk/prospero/display_record.php?ID=CRD42021246417.

### 2.1. Database Search Method

The medical librarian (JYK) developed comprehensive searches on 27 October 2020 in the following databases: MEDLINE (via Ovid), Embase (Ovid), Cochrane Library (Wiley), CINAHL, Scopus, and ProQuest Dissertations and Theses Global. Search strategies included keywords and controlled vocabulary related to augmented renal clearance in critical care (Supplementary Table S1). There were no date or language limits applied. To better facilitate the screening process, the research team used Covidence, a web-based systematic review screening tool (www.covidence.org). In addition to subscription databases, the first 200 results from Google Scholar were evaluated for inclusion. Bibliographies from included studies were also reviewed.

### 2.2. Inclusion and Exclusion Criteria

We included human studies conducted in critically ill adult populations that reported ARC prevalence and/or risk factors in our analysis. Studies also needed to have a clearly defined criteria for ARC and reported what method was used to measure or calculate CrCl. We excluded studies that focused on pediatric patients or patients with renal dysfunction (e.g., acute kidney injury), as well as studies conducted in populations that would have altered renal elimination (e.g., cystic fibrosis, burn patients). Case reports, reviews, editorials and commentaries were also excluded.

### 2.3. Study Screening

Study screening and selection were conducted independently by SHM and AS using Covidence. This was completed in two steps: (1) An initial title and abstract screening was performed. (2) The relevant abstracts were then introduced to a full-text review. The authors used discussion to come to a consensus about any arising conflicts during the screening process. Non-English language studies were translated using the Google Translate web-based document translator, when possible.

### 2.4. Data Extraction

The data were extracted independently by AS and FH from each of the included studies and then cross-checked to verify the integrity and completeness of the information. Any inconsistencies were resolved by discussion with SHM. The extracted data included: study design, exclusion and inclusion criteria, intensive care unit (ICU) type, ARC definition, diagnoses, patient demographics and ARC prevalence and risk factors contributing to ARC along with their measures of association. For studies that did not specify a cut-off for ARC but reported individual CrCl values, a value of >130 mL/min/1.73 m^2^ was applied to determine ARC prevalence. 

### 2.5. Risk of Bias Assessment

All the included studies were individually assessed for their risk of bias by employing the “Joanna Briggs Institute Critical Appraisal Instrument for Studies Reporting Prevalence Data” (https://jbi.global/sites/default/files/2020-08/Checklist_for_Prevalence_Studies.pdf). This critical appraisal tool assessed nine aspects to assess the quality of each study: (1) Was the sample frame appropriate to address the target population? (2) Were study participants sampled in an appropriate way? (3) Was the sample size adequate? (4) Were the study subjects and the setting described in detail? (5) Was the data analysis conducted with sufficient coverage of the identified sample? (6) Were valid methods used for the identification of the condition? (7) Was the condition measured in a standard, reliable way for all participants? (8) Was there appropriate statistical analysis? (9) Was the response rate adequate, and if not, was the low response rate managed appropriately? 

### 2.6. Data Analysis

The statistical analysis was performed by FH in consultation with a biostatistician using the package in R Statistical Software (Version 4.0.3, R Foundation for Statistical Computing, Vienna, Austria) and RStudio Interface (Version 1.3.1093, RStudio, Boston, MA, USA) [20,21,22]. For the meta-analysis of prevalence, the function metaprop was used to pool the meta-analytic estimate of prevalence of ARC using the reported number of cases and the total number of subjects in each included trial. We generated a random-effects meta-analytic model using the inverse variance method for weights, DerSimonian-Laird estimator [23,24] for Ƭ^2^ as the measure of true between-study variance, the Jackson method for confidence interval of Ƭ^2^ [25] and a Logit transformation to the calculated individual studies prevalence. Additionally, we examined the I^2^ statistic (the estimate of residual heterogeneity that is not due to sampling variation alone) and Cochrane Q statistic (describes the total heterogeneity not stemming from random error). The analyses were then visualized graphically using forest plots. To assess the risk of publication bias, Egger’s test [26] was conducted and tested for significance; a funnel plot was used to visualize the individual studies’ effect sizes against their estimate of precision. For studies reporting data for more than one distinct patient population, each population was entered separately in the meta-analysis. For the meta-analysis of risk factors, the function “metagen” from the package “meta” in R was utilized. It was used to synthesize the meta-analytic odds ratio size of the commonly reported risk factors: age, male sex, trauma, sequential organ failure assessment (SOFA) score, acute physiology and chronic health evaluation (APACHE II), and diabetes on ARC from their reported odds ratios of multivariate logistic regression.

## 3. Results

**Figure 1:**
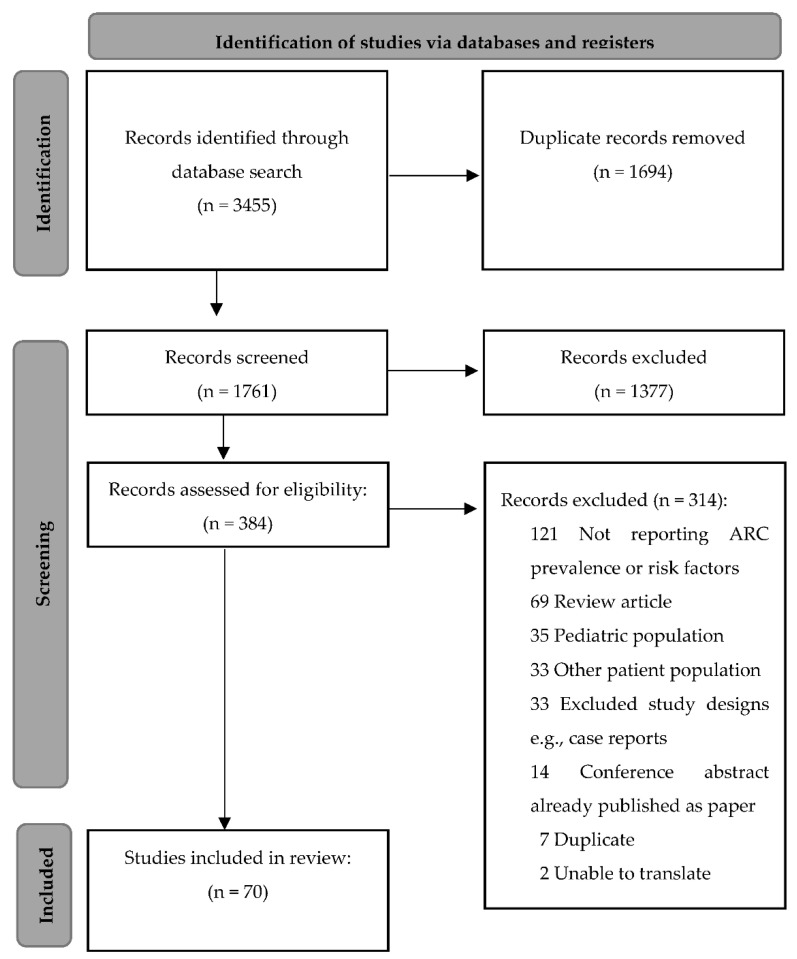
Flow chart of the study search and screening.

As depicted in Figure 1, comprehensive searches identified 3455 records across all databases. A total of 1761 records remained for screening after the removal of duplicate records. After the title and abstract screening, 384 records were subject to a full-text screening ending with a total of 70 included records Observational studies constituted the majority of collected evidence at 68 studies, along with 1 randomized controlled trial [27] and 1 prospective non-randomized interventional study [28]. Table 1 depicts a summary of the studies included in this systematic review and meta-analysis of prevalence and risk factors. Table 2 depicts a summary of the studies reporting other risk factors not included in the meta-analysis. Supplementary Table S2 depicts the risk of bias assessment of the included studies using the Joanna Briggs Institute critical appraisal tool for studies reporting prevalence data. The average score of all studies was 94.4%.

### 3.1. ARC Definition

Of the 70 included studies, 68 studies reported prevalence data. Studies varied in their definition of ARC in terms of CrCl cut-off. Most studies (52 records (76.5%)) defined ARC as CrCl ≥ 130 mL/min/1.73 m^2^; other definitions used were CrCl ≥ 120 mL/min/1.73 m^2^ (9 records (13.2%)), CrCl ≥ 150 mL/min/1.73 m^2^ (3 records (4.4%)), CrCl ≥ 140 mL/min/1.73 m^2^ (1 record (1.5%)), CrCl ≥ 155 mL/min/1.73 m^2^ (1 record (1.3%)), CrCl ≥ 160 mL/min/1.73 m^2^ (1 record (1.5%)), and CrCl ≥ 108 mL/min/1.73 m^2^ (1 record (1.5%)).

### 3.2. ARC Prevalence

Reports on the prevalence of ARC in this meta-analysis ranged between 4% and 100% in various critically ill populations, with an interquartile range of 25.9–55.8%, which suggests that ARC occurs very commonly. Our meta-analysis of prevalence included 68 studies representing 76 samples: 29 (38.2%) from mixed ICUs, 14 (18.4%) from sepsis ICUs, 9 (11.8%) from neuro ICUs, 9 (11.8%) from trauma ICUs, and 15 (19.7%) including patients from surgical, oncology, and other critically ill and non-critically ill hospitalized patients (Table 1). CrCl determination methods varied among studies, where 52 (68.4%) studies measured CrCl utilizing a 6–24 h urine collection method and 24 (31.6%) studies calculated CrCl using various equations. Among the studies that calculated CrCl, the majority used Cockcroft and Gault’s formula (*n* = 15). 

**Figure 2:**
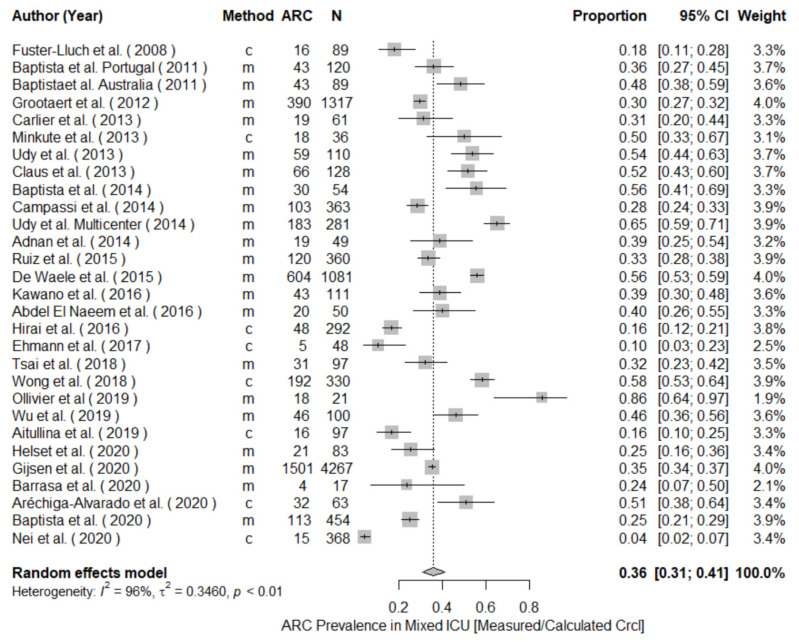
Forest plot of the prevalence of ARC in mixed intensive care unit (ICU) population. Clearance Determination method: m = measured, c = calculated; CI, confidence interval; N, study size.

**Figure 3:**
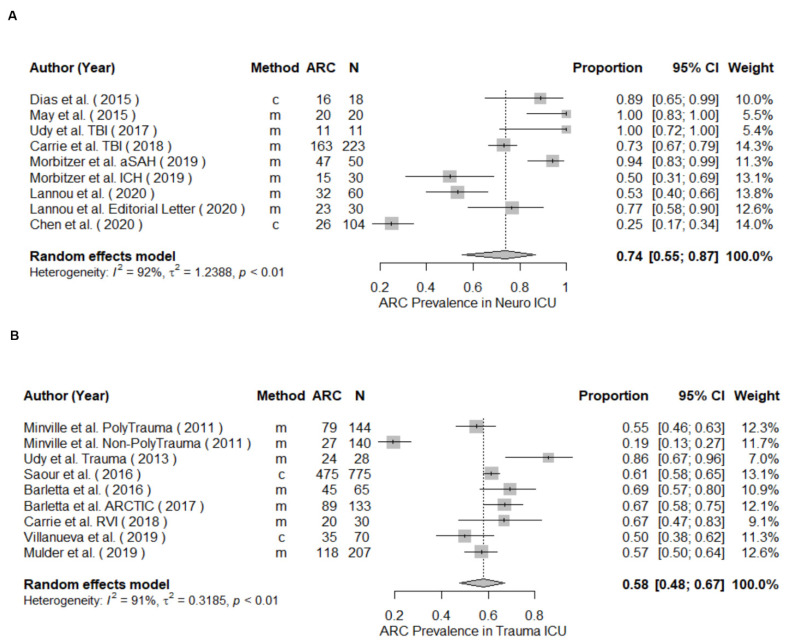
Forest plot of the prevalence of ARC in neurocritical care (**A**) and trauma intensive care unit (ICU) population (**B**). Clearance Determination method: m = measured, c = calculated; CI, confidence interval; N, study size.

**Figure 4:**
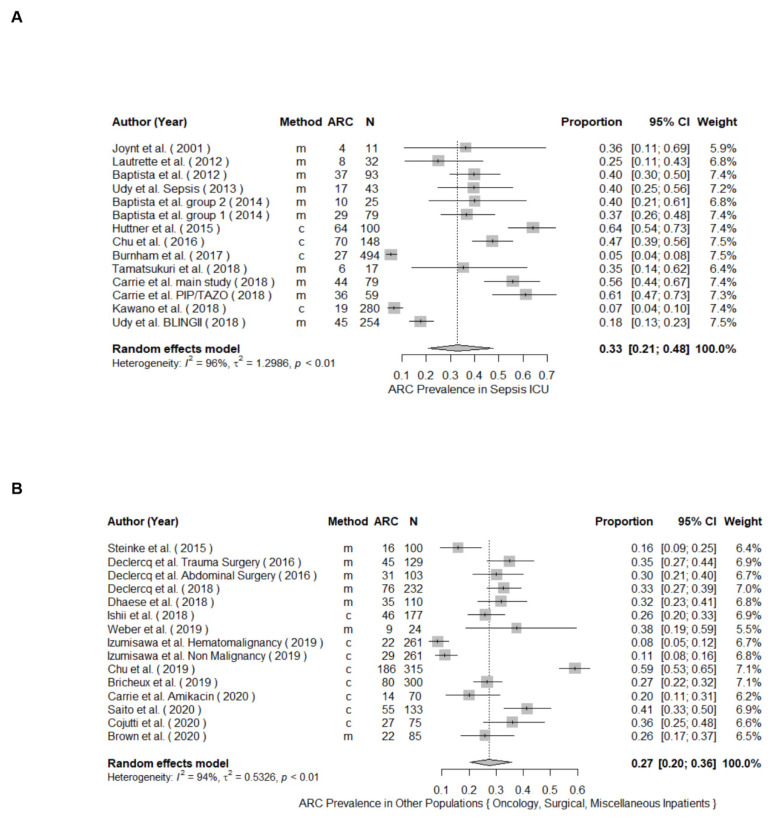
Forest plot of the prevalence of ARC in sepsis intensive care unit (ICU) (**A**) and other population (**B**). Clearance Determination method: m = measured, c = calculated; CI, confidence interval; N, study size.

**Figure 5:**
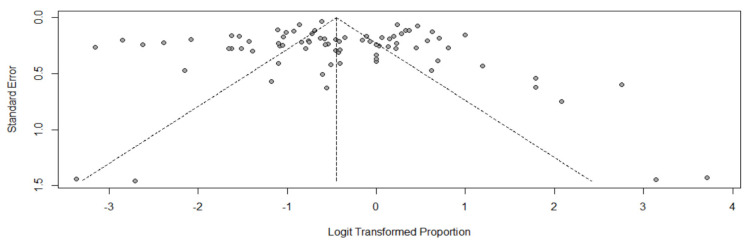
Funnel plot of studies reporting prevalence.

The meta-analysis of prevalence of all included studies yielded a pooled prevalence (95% CI) of 39% (34.9–43.3) including patients from mixed (Figure 2), neuro, sepsis, trauma, surgical, and oncology critical care units, as well as non-ICU patients. The highest ARC occurrence was detected in neurocritical care patients with a 74% pooled prevalence across the 9 studies (Figure 3A), followed by 58% in trauma ICUs across 9 studies (Figure 3B), 36% in mixed ICUs across 29 studies (Figure 2), 33% in sepsis ICUs (Figure 4A), and 27% in the other patient populations collectively (Figure 4B). A meta-analysis of ARC prevalence in studies that only measured CrCl yielded a prevalence of 41% (35–46), while, in studies that calculated mathematical estimates of CrCl, the pooled prevalence was 23% (11–43), showing a stark underestimation in the case of calculated CrCl (Supplementary Figure S1). To assess the risk of publication bias, a funnel plot was used to visualize the individual studies’ effect sizes against their estimate of precision (Figure 5). Egger’s test [26] was conducted to test for funnel plot’s asymmetry; the result was insignificant (*p*-value > 0.05), suggesting no publication bias.

### 3.3. ARC Risk Factors

**Figure 6:**
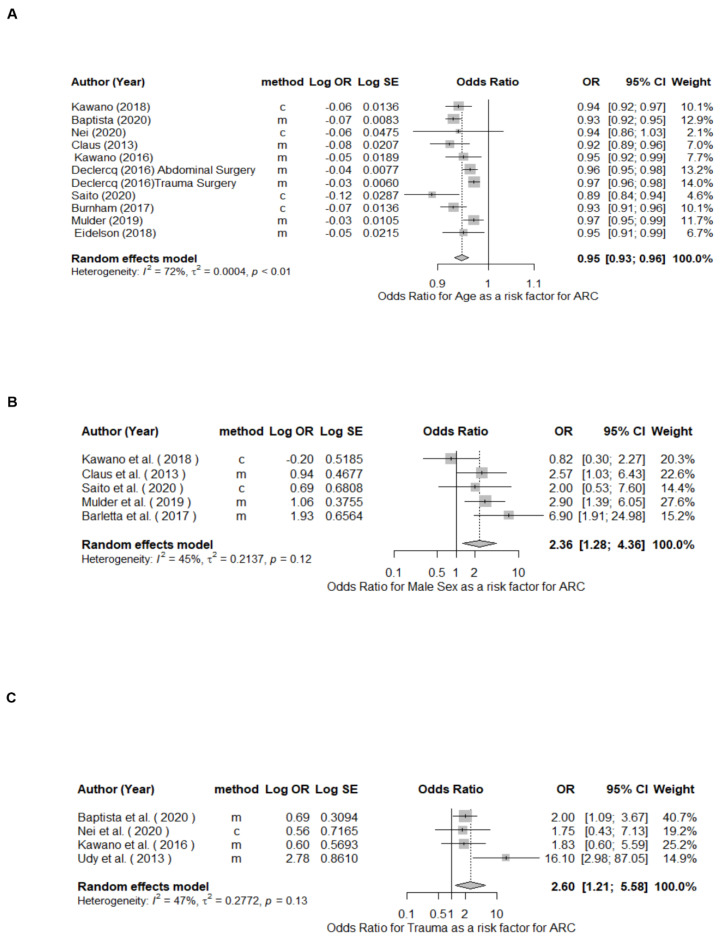
Forest plot of risk factors for augmented renal clearance. (**A**), age (as continuous variable); (**B**), male sex; (**C**), trauma. Clearance Determination method: m = measured, c = calculated; CI, confidence interval; OR, odds ratio; SE, standard error.

Reported risk factors included in the meta-analysis were age (as a continuous variable), male sex, trauma, SOFA and APACHEII disease severity scores, and diabetes. Among the reported risk factors, age, male sex and trauma were significantly associated with ARC with pooled odds ratio (95% CI) estimates of 0.95 (0.93–0.96), 2.36 (1.28–4.36), and 2.60 (1.21–5.58), respectively (Figure 6). SOFA, APACHEII and diabetes were not significantly associated with ARC, with pooled odds ratio (95% CI) estimates of 0.86 (0.73–1.01), 1.00 (0.95–1.06) and 1.21 (0.46–3.17), respectively (Supplementary Figure S2). 

## 4. Discussion

ARC is a phenomenon wherein renal clearance is accelerated beyond normal range; it has also been referred to as glomerular hyperfiltration or enhanced renal clearance. ARC bears the risk of causing therapeutic failure of predominantly renally cleared drugs, which could be especially detrimental in critically ill populations. Numerous studies have described the association between ARC and higher rates of failure to attain therapeutic levels and compromised effectiveness of various drugs and the need for a more frequent administration and/or higher dosages. Standard doses of renally-eliminated medications are typically used in patients with “normal” renal function. However, pharmacodynamic targets that are consistently obtained in other populations with typical dosing are not met in the presence of ARC. Studies have suggested that ARC might be associated with subtherapeutic concentrations of antimicrobials and AEDs, [33,77,78,91] antimicrobial therapy failure, [40] increased odds of recurrent infections, [18] and poor seizure control [92]. Our systematic review and meta-analysis demonstrated the common occurrence of ARC in critical care settings, with higher prevalence among neurocritical care and trauma patients compared to mixed ICU population. In addition, risk factors consistently found to be associated with ARC includes age, male sex, and trauma. The differences in the pooled ARC prevalence demonstrated that different critically ill populations were not at an equivalent risk for ARC and highlighted the importance of screening for ARC in select patient populations, as well as the need to develop new screening tools that account for these risk differences. To our knowledge, this is the first combined systematic review and meta-analysis of the prevalence and risk factors of ARC. 

In our random effects meta-analysis for ARC prevalence, patients in the neurocritical care population demonstrated the highest prevalence of ARC (74%). ARC incidence has been reported to range much higher in neurocritical care patients compared to the general critically ill population [4,11,12,13,14,15,16,17,18]. To illustrate, in a study of 20 traumatic brain injury (TBI) patients, 85% showed ARC [14]. In a study of patients with hemorrhagic stroke, ARC was reported in 50% of intracerebral hemorrhage (ICH) (n = 30) and 94 % of subarachnoid hemorrhage (n = 50) patients [16]. In addition, ICH was found to predict ARC in a retrospective study of heterogenous ICU patients, supporting the notion that neurological injury poses additional ARC risk [88]. This could be attributed to the possibility that patients with neurological injuries might have additional ARC risks. Neurocritical care patients tend to be relatively younger patients with single comorbidities and otherwise unimpaired organ systems, as well as a lower incidence of renal impairment. Furthermore, neurological injury could play an additional role in the pathophysiology of ARC; however, further studies are needed to confirm such association [47,61].

The employment of an accurate determination method for glomerular filtration rate is essential for ARC screening and diagnosis. Although using serum creatinine to assess kidney function carries limitations, CrCl measurement using 8-24h urine collection is the most agreed upon accurate method for the measurement of renal function in the clinical setting without the need of administrating an exogenous substance such as inulin. Moreover, due to the impracticality of routine and frequent measurement of CrCl in clinical settings, calculating CrCl using mathematical estimations derived from population parameters is often employed to allow for a more rapid determination. Commonly used formulae used to draw mathematical estimates of CrCl include the Cockcroft–Gault equation (CG), modification of diet in renal diseases (MDRD), and chronic kidney disease-epidemiology (CKD-EPI). Each of those methods possess their own merits and downfalls. Several studies assessed the relative accuracy of different mathematical estimates of CrCl in patients exhibiting ARC. It has been found that all mathematical estimations of CrCl grossly underestimate the actual CrCl when compared with their respective measured CrCl in patients with ARC [31,38,41,44,45,49,54,93,94,95]. Similarly, we found that the mathematical estimations of CrCl grossly underestimated the prevalence in ARC when compared to measured CrCl. To illustrate, the meta-analysis of prevalence of ARC in the same population (mixed ICU patients) was 23% in studies using mathematical estimates, whereas studies using measured CrCl showed a 41% prevalence. Therefore, we recommend obtaining a patient’s measured CrCl at least once on admission for a more judicious assessment if they are at risk for ARC. Special consideration must also be taken in immobile patients, children, burn patients or patients with conditions causing lower muscle mass or amputations to account for the reduced production of creatinine in these cases which could result in falsely low serum creatinine levels leading to incorrect diagnosis of augmented renal clearance.

It has been consistently shown in studies reporting risk factors of ARC that ARC patients tend to be younger males (<50 years old) with lower critical illness severity scores. These patients also tend to suffer from single organ impairment with unimpaired kidney function and a history of recent trauma. In our analysis, among the reported risk factors, age, male sex, and trauma were significantly associated with ARC with pooled odds ratio (95% CI) estimates of 0.95 (0.93–0.96), 2.36 (1.28–4.36), and 2.60 (1.21–5.58), respectively. The aforementioned risk factors have been utilized to develop clinical prediction tools needed for the early identification of patients at a higher risk for developing ARC. An ARC scoring system with 60% sensitivity and 95% specificity was introduced by Baptista et al. [96], where urinary creatinine higher than 45 mg/mL, age less than 65 years, and blood urea nitrogen (BUN) less than 7 mmol/L serve as predictors of ARC. Moreover, Udy et al. developed a scoring system that is based on age less than 50 years old, history of recent trauma, and SOFA score ≤ 4 [36]. This tool demonstrated 100% sensitivity and 71% specificity when validated by Akers et al. [97]. Furthermore, Barletta et al. [60] developed the augmented renal clearance in trauma intensive care (ARCTIC) scoring system, which eliminated the need to calculate a SOFA score in order to assess the patients’ risk for developing ARC, which can be impractical in some patient settings. The risk factors employed in the assessment tool were serum creatinine, sex and age; it stratified patients into high risk (ARCTIC score ≥ 6) and low risk (ARCTIC score < 6). Employing predictive tools such as ARC or ARCTIC in routine screening of critically ill patients could be valuable in the way of early recognition and timely management of ARC patients. However, the developed scoring tools were generated based on the general critically ill/trauma population rather than patients with severe neurological illnesses, potentially not capturing neurocritical care patients with additional risks for ARC. 

Our systematic review was limited by the characteristics of the included studies. The main body of evidence comes from retrospective observational studies, which require caution in the interpretation of results. In addition, heterogeneity of the included studies was high secondary to variations in study populations, ARC definitions, the method of determining CrCl, studies inclusion and exclusion criteria may impede accurate comparisons among studies. For example, 65% of the studies in the meta-analysis excluded patients with existing acute and/or chronic renal impairment with various stages, impeding the possibility of extrapolating their results outside of the sampling context, as well as overestimating ARC occurrence in these samples compared to others where patients with renal impairment were included [9,18,59,88]. This highlights the need for unified assessment of ARC in future research. However, in our analysis, we took into consideration the heterogeneity of the included studies; our pooled estimates are a reasonable representation of the body of literature. 

## 5. Conclusions

ARC is a prevalent phenomenon in critically ill patients especially neurocritical care and trauma ICU population. Young age, male sex, and trauma are risk factors for ARC in those with apparently normal renal function. The estimation of CrCl using mathematical estimates of GFR grossly underestimates the prevalence of ARC in the critical care setting; therefore measured CrCl through urine collections is prudent. Further research on optimal dosing of drugs in the setting of ARC is warranted.

## Figures and Tables

**Table 1:** Summary characteristics of studies included in ARC systematic review and meta-analysis of prevalence and risk factors.

Author	Year	Population	Study Design	Clearance Determination		ARC Definition	N	Prevalence (%)	Male n (%)	Age *	Main Diagnoses	Identifiable Risk Factors	Renal Impairment
Joynt et al. [29]	2001	Sepsis ICU	prospective observational	m	24 h Urine	130	11	36.4	7 (63.6)	45 ± 16	Sepsis	not reported	Excluded
Fuster-Lluch et al. [30]	2008	Mixed ICU	prospective observational	c	NKF	120	89	18.0	67 (75.3)	60.5 (18–86)	Several	not reported	Excluded
Baptista et al. Portugal [31]	2011	Mixed ICU	prospective observational	m	24 h Urine	130	120	35.8	87 (72.5)	55.9 ± 21.1	Sepsis, Trauma	not reported	Excluded
Baptista et al. Australia [31]	2011	Mixed ICU	prospective observational	m	8 h Urine	130	89	48.3	64 (71.9)	40 ± 18.9	Sepsis, Trauma	not reported	Excluded
Minville et al. PolyTrauma [32]	2011	Trauma ICU	retrospective observational	m	24 h Urine	120	144	54.9	108 (75)	42 ± 18	Poly trauma ICU	Age Trauma	Excluded
Minville et al. Non-PolyTrauma [32]	2011	Trauma ICU	retrospective observational	m	24 h Urine	120	140	19.3	88 (62.8)	58 ± 17	Non trauma ICU	Age Trauma	Excluded
Lautrette et al. [17]	2012	Sepsis ICU	retrospective observational	m	24 h Urine	140	32	25.0	15 (46.8)	54 ± 16	Infectious meningitis	not reported	Included
Baptista et al. [33]	2012	Sepsis ICU	prospective observational	m	24 h Urine	130	93	39.8	69 (74.2)	58 (34–75)	Trauma, Sepsis, Other.	not reported	Excluded
Grootaert et al. [34]	2012	Mixed ICU	retrospective observational	m	24 h Urine	120	1317	29.6	247 (18.8)	59 (48–67)	Several	not reported	Unclear
Carlier et al. [35]	2013	Mixed ICU	prospective observational	m	24 h Urine	130	61	31.1	51 (85)	56 (48–67)	Infections	not reported	Excluded
Udy et al. Sepsis [36]	2013	Sepsis ICU	prospective observational	m	6 h Urine	130	43	39.5	22 (51.2)	46.3 ± 17.1	Sepsis	Age, Trauma, mod. SOFA	Included
Udy et al. Trauma [36]	2013	Trauma ICU	prospective observational	m	6 h Urine	130	28	85.7	23 (82.1)	36.4 ± 13.9	Trauma	Age, Trauma, mod. SOFA	Included
Minkute et al. [37]	2013	Mixed ICU	retrospective observational	c	C&G	130	36	50.0	29 (80.5)	49.75 (21)	Several	not reported	Excluded
Udy et al. [38]	2013	Mixed ICU	prospective observational	m	8 h Urine	120	110	53.6	70 (63.6)	50.9 ± 16.9	Several	not reported	Excluded
Claus et al. [39]	2013	Mixed ICU	prospective observational	m	24 h Urine	130	128	51.6	86 (67.2)	59 (49–67.8)	Several	Age, APACHEII, Male sex	Excluded
Baptista et al. group 2 [40]	2014	Sepsis ICU	prospective observational	m	8 h Urine	130	25	40.0	17 (68)	59.9 ± 17.2	Several	not reported	Excluded
Baptista et al. group 1 [40]	2014	Sepsis ICU	retrospective observational	m	8 h Urine	130	79	36.7	52 (66)	57.8 ± 15.5	Several	not reported	Excluded
Baptista et al. [41]	2014	Mixed ICU	prospective observational	m	8 h Urine	130	54	55.6	39 (72.2)	54.2 ± 16.9	Several	not reported	Excluded
Campassi et al. [42]	2014	Mixed ICU	prospective observational	m	24 h Urine	120	363	28.4	103 (28.4)	56.5 ± 16	Several	Age, DM	Excluded
Udy et al. Multicenter [43]	2014	Mixed ICU	prospective observational	m	8 h Urine	130	281	65.1	178 (63.3)	54.4 (52.5–56.4)	Several	not reported	Excluded
Adnan et al. [44]	2014	Mixed ICU	prospective observational	m	24 h Urine	130	49	38.8	37 (75.5)	34 (24–47)	Trauma, others	not reported	Excluded
Ruiz et al. [45]	2015	Mixed ICU	prospective observational	m	24 h Urine	130	360	33.3	246 (68.3)	50 ± 19	Polytrauma, Non-polytrauma	Age, Polytrauma	Excluded
Huttner et al. [46]	2015	Sepsis ICU	prospective observational	c	C&G	130	100	64.0	75 (73.5)	46 ± 10.55	Several	not reported	Excluded
Dias et al. [47]	2015	Neuro ICU	retrospective observational	c	C&G	130	18	88.9	16 (89)	41 ± 15.6	TBI, Polytrauma	not reported	Included
May et al. [15]	2015	Neuro ICU	prospective observational	m	24 h Urine	130	20	100.0	8 (40)	52.14 ± 10.36	SAH	not reported	Excluded
De Waele et al. [48]	2015	Mixed ICU	retrospective observational	m	24 h Urine	130	1081	55.9	687 (63.6)	62 (20.5)	Several	not reported	Excluded
Steinke et al. [49]	2015	Surgical ICU	retrospective observational	m	18 h Urine	130	100	16.0	61 (61)	66 (57–74)	Infection, others	not reported	Included
Chu et al. [50]	2016	Sepsis ICU	retrospective observational	c	C&G	130	148	47.3	97 (65.5)	55.3 ± 14.9	Infection	not reported	Excluded
Kawano et al. [51]	2016	Mixed ICU	prospective observational	m	8 h Urine	130	111	38.7	62 (55.9)	67 (53–770)	Several	Age, DM, Weight, APACHEII, others	Excluded
Saour et al. [52]	2016	Trauma ICU	retrospective observational	c	MDRD	120	775	61.3	581 (75)	37.7 ± 17	Several	not reported	Excluded
Abd El Naeem et al. [53]	2017	Mixed ICU	prospective observational	m	24 h Urine	130	50	40.0	32 (64)	71 ± 15	Sepsis, others	not reported	Excluded
Barletta et al. [54]	2016	Trauma ICU	retrospective observational	m	12 h Urine	130	65	69.2	48 (74)	48 ± 18	TBI, other traumas	not reported	Unclear
Declercq et al. Trauma Surgery [55]	2016	Surgical non-ICU	prospective observational	m	8 h Urine	130	129	34.9	75 (58)	62 (46–75)	Trauma surgery	Age, Sex	Excluded
Declercq et al. Abdominal Surgery [55]	2016	Surgical non-ICU	prospective observational	m	8 h Urine	130	103	30.1	76 (74)	63 (51–71)	Abdominal surgery	Age	Excluded
Hirai et al. [3]	2016	Mixed ICU	retrospective observational	c	C&G	130	292	16.4	185 (63.4)	72 (62.8–82)	Several	Age, Brain injury, others	Excluded
Ehmann et al. [56]	2017	Mixed ICU	prospective observational	c	C&G	130	48	10.4	27 (56.3)	55.5 (32–69.9)	Sepsis, others	not reported	Included
Burnham et al. [57]	2017	Sepsis ICU	retrospective observational	c	MDRD	130	494	5.5	260 (52.6)	59.9 ± 15.8	Sepsis	Age, sepsis severity, others	Included
Carrie et al. RVI [58]	2018	Trauma ICU	retrospective observational	m	24 h Urine	130	30	66.7	27 (90)	48 (32–67)	Polytrauma, TBI	not reported	Excluded
Udy et al. TBI [59]	2017	Neuro ICU	prospective observational	m	8 h Urine	150	11	100.0	9 (81.8)	37 (24–49)	TBI	not reported	Included
Barletta et al. ARCTIC [60]	2017	Trauma ICU	prospective observational	m	12 h Urine	130	133	66.9	101 (76)	48 ± 19	TBI, fractures, others	Age, Sex	Excluded
Dhaese et al. [61]	2018	Surgical ICU	prospective observational	m	8 h Urine	130	110	31.8	75 (68.2)	60 ± 14.4	Several	not reported	Excluded
Tamatsukuri et al. [62]	2018	Sepsis ICU	prospective observational	m	8 h Urine	130	17	35.3	11 (64.7)	60 (19.5)	Sepsis	not reported	Excluded
Carrie et al. main study [2]	2018	Sepsis ICU	prospective observational	m	24 h Urine	150	79	55.7	62 (78)	52 (33–68)	Sepsis	not reported	Excluded
Carrie et al. PIP/TAZO [63]	2018	Sepsis ICU	prospective observational	m	24 h Urine	130	59	61.0	47 (80)	53 ± 21	Polytrauma, non-trauma surgery	not reported	Excluded
Carrie et al. TBI [18]	2018	Neuro ICU	prospective observational	m	24 h Urine	130	223	73.1	184 (83)	36 (23–57)	TBI, VAP	not reported	Included
Kawano et al. [64]	2018	Sepsis ICU	retrospective observational	c	Japanese equation	130	280	6.8	145 (51.8)	74 (64–83)	Infection	Age, Sex, DM, others	Excluded
Tsai et al. [65]	2018	Mixed ICU	prospective observational	m	8 h Urine	130	97	32.0	60 (46)	50 ± 18	Sepsis, Trauma, others	not reported	Excluded
Wong et al. [66]	2018	Mixed ICU	prospective observational	c	C&G	130	330	58.2	198 (60)	53.4 ± 17.7	Infection	not reported	Included
Ishii et al. [67]	2018	Mixed ICU—Non-ICU	retrospective observational	c	Japanese equation	120	177	26.0	109 (62)	73 (63–80)	Tumors, Brain injury	not reported	Excluded
Udy et al. BLINGII [27]	2018	Sepsis ICU	randomized controlled trial	m	8 h Urine	130	254	17.7	151 (59.4)	63 (52–71)	Infection	not reported	Included
Ollivier et al. [68]	2019	Mixed ICU	prospective observational	m	24 h Urine	150	21	85.7	17 (81)	36 (27–60)	Trauma, Surgery	not reported	Included
Wu et al. [69]	2019	Mixed ICU	prospective observational	m	24 h Urine	130	100	46.0	66 (66)	60 (47–71)	Several	Age, SOFA, Weight, others	Excluded
Aitullina et al. [70]	2019	Mixed ICU	retrospective observational	c	not reported	108	97	16.5	65 (67)	63 (51–73.5)	Several	not reported	Included
Weber et al. [71]	2019	Oncology ICU	prospective observational	m	24 h Urine	120	24	37.5	14 (58.3)	59 (39.8–63.5)	Febrile neutropenia	not reported	Excluded
Izumisawa et al. Hematomalignancy [72]	2019	Oncology Non-ICU & ICU	retrospective observational	c	C&G	120	261	8.4	146 (55.9)	65.6 ± 13.6	Hematologic malignancy	not reported	Excluded
Izumisawa et al. Non-Malignancy [72]	2019	Oncology Non-ICU & ICU	retrospective observational	c	C&G	120	261	11.1	175 (67)	67.2 ± 16.9	Non malignancy	not reported	Excluded
Chu et al. [73]	2019	Mixed ICU—Non-ICU	retrospective observational	c	C&G	130	315	59.0	213 (67.6)	56.3 (19)	Infection	not reported	Excluded
Villanueva et al. [74]	2019	Trauma ICU	retrospective observational	c	C&G	160	70	50.0	57 (81.4)	47.5 (31–61)	TBI, Spinal injury	not reported	Excluded
Morbitzer et al^.^ aSAH [75]	2019	Neuro ICU	prospective observational	m	8 h Urine	130	50	94.0	16 (32)	57.2 ± 10.7	SAH	not reported	Excluded
Morbitzer et al. ICH [75]	2019	Neuro ICU	prospective observational	m	8 h Urine	130	30	50.0	18 (60)	70 ± 13.7	ICH	not reported	Excluded
Mulder et al. [76]	2019	Trauma ICU	retrospective observational	m	24 h Urine	130	207	57.0	141 (68)	45 ± 20	Trauma	Age, Sex, others	Excluded
Bricheux et al. [77].	2019	Hospitalized	retrospective observational	c	C&G	130	300	26.7	203 (68)	59 ± 17	Abdominal infection, Pneumonia	not reported	Unclear
Helset et al. [78]	2020	Mixed ICU	prospective observational	m	24 h Urine	130	83	25.3	61 (73.5)	54.5 (38–63)	Several	not reported	Unclear
Gijsen et al. [7]	2020	Mixed ICU	retrospective observational	m	24 h Urine	130	4267	35.2	2669 (62.5)	65 (54–74)	Several	not reported	Excluded
Barrasa et al. [79]	2020	Mixed ICU	prospective observational	m	10 h Urine	130	17	23.5	12 (70.6)	61.7	Several	not reported	Included
Lannou et al. [80]	2020	Neuro ICU	prospective observational	m	24 h Urine	130	60	53.3	53 (88)	48 (32–60)	TBI, Multiple trauma	not reported	Excluded
Aréchiga-Alvarado et al. [81]	2020	Mixed ICU	prospective observational	c	C&G	130	63	50.8	56 (88.9)	33.25 (47.5)	Infection	not reported	Unclear
Carrie et al. Amikacin [82]	2020	Surgical ICU	retrospective observational	c	C&G	130	70	20.0	53 (76)	65 (51–73)	Infection	not reported	Unclear
Saito et al. [83]	2020	Oncology ICU	retrospective observational	c	own predictive model	130	133	41.4	80 (60.2)	64 (25–86)	Haematologic malignancies	Age, Sex, Scr, others	Included
Lannou et al. Editorial Letter [84]	2020	Neuro ICU	retrospective observational	m	24 h Urine	155	30	76.7	not reported	33 (47–57)	Brain trauma	not reported	Included
Cojutti et al. [28]	2020	Oncology ICU	prospective interventional	c	MDRD	130	75	36.0	47 (62.7)	58 (51–66)	Febrile neutropenia	not reported	Included
Brown et al. [85]	2020	Hospitalized	retrospective observational	m	8 h Urine	130	85	25.9	43 (50.6)	55 (41–70)	Several	not reported	Excluded
Chen et al. [86]	2020	Neuro ICU	retrospective observational	c	C&G	130	104	25.0	71 (68.3)	44.5 (18.5)	Cerebral tumor, Stroke, TBI	not reported	Excluded
Baptista et al. [87]	2020	Mixed ICU	retrospective observational	m	8 h Urine	130	454	24.9	293 (64.5)	66 (52–76)	Several	Age, Sex, Trauma, others	Included
Nei et al. [88]	2020	Mixed ICU	retrospective observational	c	CKD-EPI	130	368	4.1	208 (56.5)	66.8 (55.7–76.6)	TBI, Trauma, Sepsis, others	Age, ICH, SOFA, Trauma, others	Included

APACHE II = Acute Physiology and Chronic Health Evaluation; ARC = Augmented Renal Clearance; aSAH = aneurysmal subarachnoid hemorrhage; CG = Cockcroft Gault equation; CKD-EPI = Chronic Kidney Disease Epidemiology; CrCl = creatinine clearance; ICH = intracranial hemorrhage; ICU = intensive care unit; MDRD = modification of diet in renal disease method; SAH = subarachnoid hemorrhage; SAPS II = Simplified Acute Physiology Score; SCr = serum creatinine; SOFA = sequential organ failure assessment score; TBI = traumatic brain injury. * Age reported in median (IQR) or mean ± SD, ARC cut-off reported in mL/min/1.73 m^2^, Clearance Determination method: m = measured, c = calculated.

**Table 2:** Summary characteristics of individual studies reporting other risk factors.

Author	Year	Population	Sample Size	Clearance Determination	Identified Risk Factor (s)	Odds Ratio (95% CI)	Study Inclusion in Prevalence Meta-Analysis
Hirai et al. [3]	2016	Mixed Hospital	292	Calculated	Febrile Neutropenia	2.76 (1.11–6.67)	✓
					Fluid Infusion ≥ 1500 mL/day	2.53 (1.27–5.16)	
					Traumatic Brain Injury	5.11 (1.49–17.57)	
Nei et al. [88]	2020	Mixed ICU	368	Calculated	Charlson Comorbidity Index	0.80 (0.16–1.00)	✓
					Intracerebral Hemorrhage	2.82 (1–69.1)	
Kawano et al. [51]	2016	Mixed ICU	111	Measured	Post-Operative Without Sepsis	0.28 (0.07–1.04)	✓
Wu et al. [69]	2019	Mixed ICU	100	Measured	Loop Diuretics	0.32 (0.11–0.93)	✓
					Age < 50	4.02 (1.54–10.51)	
Udy et al. [36]	2013	Mixed ICU	71	Measured	Age </= 50	28.6 (4.4–187.2)	✓
Ramos et al. [89]	2017	Mixed ICU	36	Measured	24h Sodium Excretion	0.99 (0.98–1.00)	✗
Saito et al. [83]	2020	Oncology Hospital	133	Calculated	Serum Creatinine	0.89 (0.83–0.94)	✓
					Leukemia	9.4 (2.4–36.8)	
					Fever	2.4 (0.78–7.1)	
Burnham et al. [57]	2017	Sepsis ICU	494	Calculated	African American Ethnicity	3.45 (1.40–8.50)	✗
					Sepsis Severity	0.54 (0.30–0.97)	
Mulder et al. [76]	2019	Trauma ICU	207	Measured	Packed RBC Transfusion	0.31 (0.15–0.66)	✓
Eidelson et al. [90]	2018	Trauma ICU	154	Measured	Admission Hematocrit	1.18 (1.04–1.33)	✗
Barletta et al. [60]	2017	Trauma ICU	133	Measured	Serum Creatinine < 0.7 mg/dL	12.5 (3–52.6)	✓
					Age < 56	58.3 (5.2–658.9)	
					Age 56–75	13.5 (1.2–151.7)	

## Data Availability

The data is contained within this article and the associated supplementary materials.

## Supplementary Materials

The following supporting information can be downloaded at: https://www.mdpi.com/article/10.3390/pharmaceutics14020445/s1, Table S1: Full search strategy; Table S2: Appraisal of individual studies included in this review; Figure S1: Forest plot of the prevalence of ARC in mixed intensive care unit (ICU) population. A, studies reported measured creatinine clearance (m); B, studies reported calculated creatinine clearance (c). Figure S2: Forest plot of risk factors of augmented renal clearance. A, diabetes; B, Sequential Organ Failure Assessment (SOFA) score; C, Acute Physiology and Chronic Health Evaluation (APACHE II).
