# Supplementary material for: Prevalence and Risk Factors of Augmented Renal Clearance: A Systematic Review and Meta-Analysis
Source: Pharmaceutics. 2022 Feb 19;14(2):445. doi: 10.3390/pharmaceutics14020445 (PMC8878755; doi:10.3390/pharmaceutics14020445)
Supplement: Supplementary file 1 [file pharmaceutics-14-00445-s001.zip › pharmaceutics-1557467-supplementary.pdf]

Supplementary Materials: Prevalence and Risk Factors of Augmented Renal Clearance: A Systematic Review and Meta-Analysis

Fatma Hefny, Anna Stuart, Janice Y. Kung and Sherif Hanafy Mahmoud

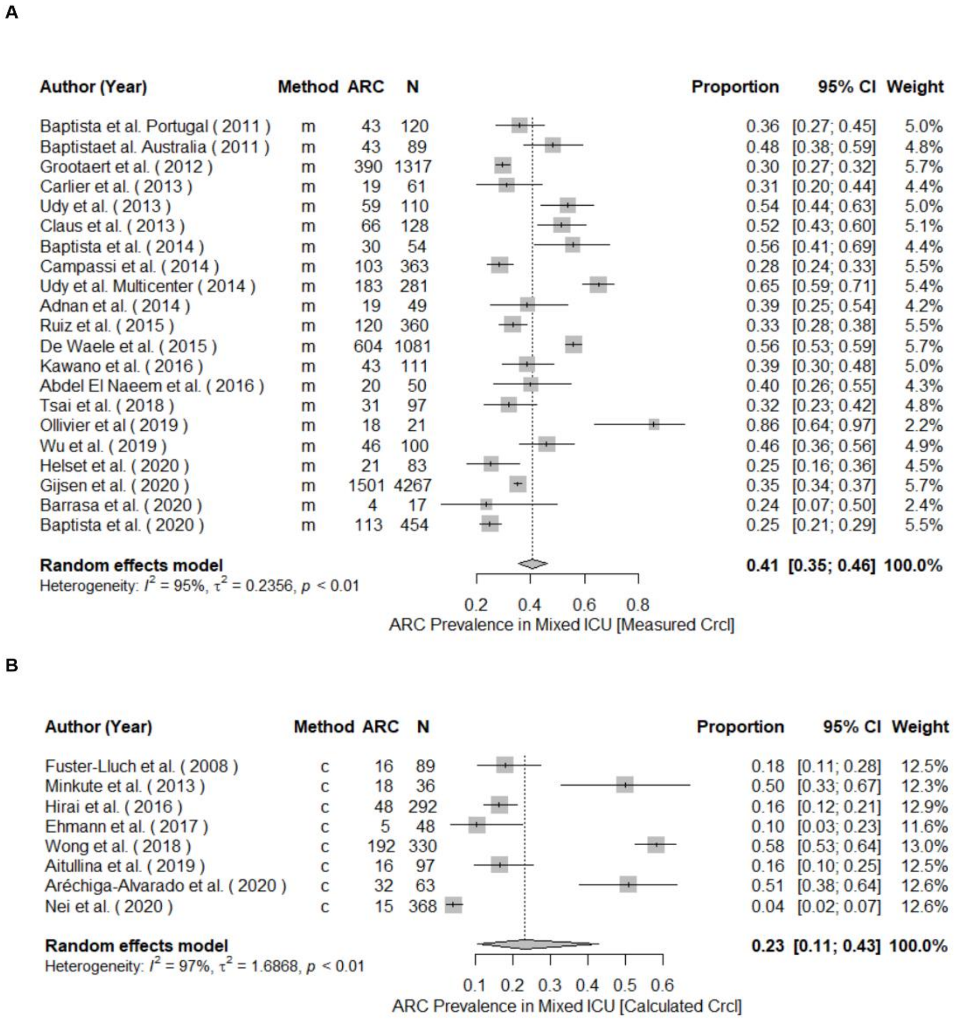

Figure S1. Forest plot of the prevalence of ARC in mixed intensive care unit (ICU) population. **A**, studies reported measured creatinine clearance (m); **B**, studies reported calculated creatinine clearance (c).

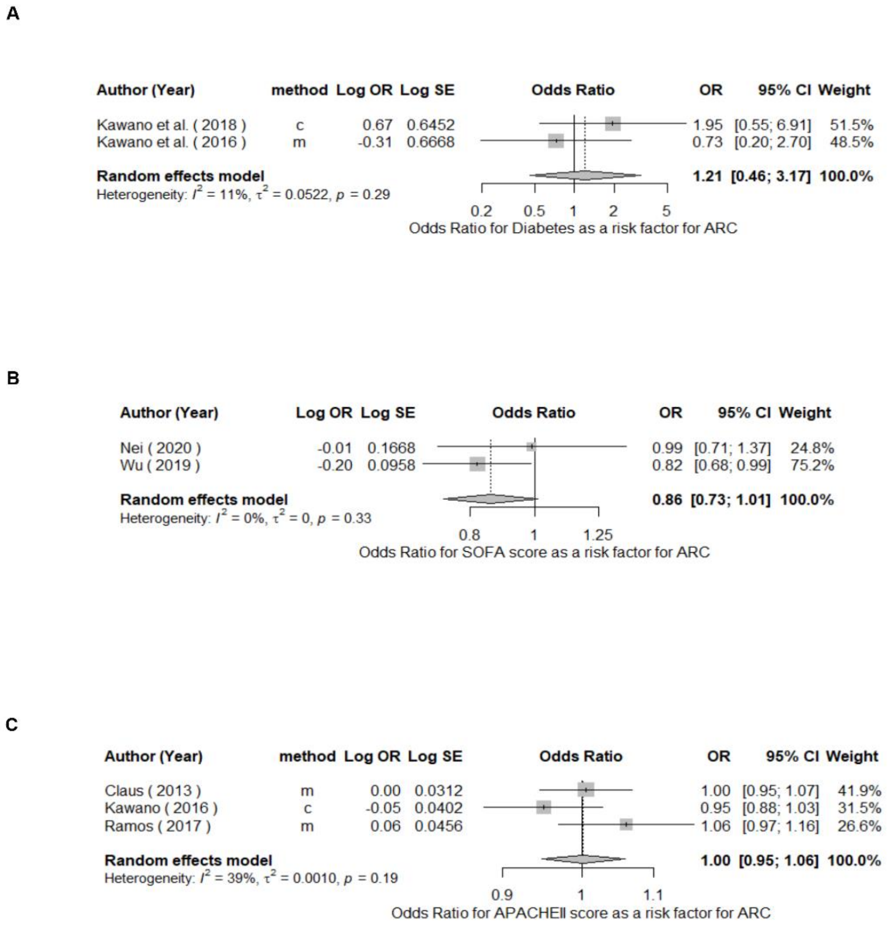

Figure S2. Forest plot of risk factors of augmented renal clearance. **A**, diabetes; **B**, Sequential Organ Failure Assessment (SOFA) score; **C**, Acute Physiology and Chronic Health Evaluation (APACHE II).

Prevalence and Risk Factors of Augmented Renal Clearance: A Systematic Review and Meta-analysis

Table S1. Full search strategy.

Database	Search Strategy
**MEDLINE**<br><br>**Ovid MEDLINE(R) ALL** 1946 to October 26, 2020	1. augmented renal clearance.mp.<br>2. augmented kidney clearance.mp.<br>3. ((increas* or enhanc* or high*) adj3 (kidney or renal) adj1 (function or clearance)).mp.<br>4. ((increas* or high*) adj3 (creatinine clearance or drug clearance or med* clearance)).mp.<br>5. (ultrafiltrat* adj3 (kidney or renal)).mp.<br>6. glomerular hyperfiltration.mp.<br>7. 3 or 4 or 5 or 6<br>8. exp *Intensive Care Units/<br>9. (ICU or intensive care or critical care or critical* ill* or acute care).ti,ab,kf.<br>10. exp *Critical Care/

	11. (sepsis or septic shock or trauma or brain injur* or brain bleed* or cerebral bleed* or intracerebral or intracranial or stroke* or infection* or meningitis or subarachnoid or h#emorrhag*).ti,ab,kf.<br>12. 8 or 9 or 10 or 11<br>13. 7 and 12<br>14. 1 or 2 or 13<br>15. animal/<br>16. human/<br>17. 15 not (15 and 16)<br>18. (veterinary or rabbit or rabbits or animal or animals or mouse or mice or rodent or rodents or rat or rats or hamster* or pig or pigs or porcine or horse* or equine or cow or cows or bovine or goat or goats or sheep or ovine or canine or dog or dogs or feline or cat or cats or zebrafish).ti.<br>19. 17 or 18 [animal studies]<br>20. 14 not 19<br>21. limit 20 to comment<br>22. limit 20 to editorial<br>23. 21 or 22<br>24. 20 not 23
**Embase**<br><br>**Ovid Embase** 1974 to 2020 October 26	1. augmented renal clearance.mp.<br>2. augmented kidney clearance.mp.<br>3. ((increas* or enhanc* or high*) adj3 (kidney or renal) adj1 (function or clearance)).mp.<br>4. ((increas* or high*) adj3 (creatinine clearance or drug clearance or med* clearance)).mp.<br>5. (ultrafiltrat* adj3 (kidney or renal)).mp.<br>6. glomerular hyperfiltration.mp.<br>7. 3 or 4 or 5 or 6<br>8. exp *intensive care unit/<br>9. (ICU or intensive care or critical care or critical* ill* or acute care).ti,ab,kw.<br>10. exp *intensive care/<br>11. (sepsis or septic shock or trauma or brain injur* or brain bleed* or cerebral bleed* or intracerebral or intracranial or stroke* or infection* or meningitis or subarachnoid or h#emorrhag*).ti,ab,kw.<br>12. 8 or 9 or 10 or 11<br>13. 7 and 12<br>14. 1 or 2 or 13<br>15. animal/<br>16. human/<br>17. 15 not (15 and 16)<br>18. (veterinary or rabbit or rabbits or animal or animals or mouse or mice or rodent or rodents or rat or rats or hamster* or pig or pigs or porcine or horse* or equine or cow or cows or

	bovine or goat or goats or sheep or ovine or canine or dog or dogs or feline or cat or cats or zebrafish).ti.<br>19. 17 or 18 [animal studies]<br>20. 14 not 19<br>21. limit 20 to editorial<br>22. 20 not 21
**CINAHL**	S1 augmented renal clearance<br>S2 augmented kidney clearance<br>S3 (increas* or enhanc* or high*) N2 ("kidney function" or "kidney clearance" or "renal function" or "renal clearance")<br>S4 (increas* or high*) N2 ("creatinine clearance" or "drug clearance" or "med* clearance")<br>S5 (ultrafiltrat* N3 (kidney or renal))<br>S6 "glomerular hyperfiltration"<br>S7 S3 OR S4 OR S5 OR S6<br>S8 (MM "Intensive Care Units+")<br>S9 TI (ICU or "intensive care" or "critical care" or "critical* ill*" or "acute care") OR AB (ICU or "intensive care" or "critical care" or "critical* ill*" or "acute care")<br>S10 (MM "Critical Care+")<br>S11 TI (sepsis or "septic shock" or trauma or "brain injur*" or "brain bleed*" or "cerebral bleed*" or intracerebral or intracranial or stroke* or infection* or meningitis or subarachnoid or h#emorrhag*) OR AB (sepsis or "septic shock" or trauma or "brain injur*" or "brain bleed*" or "cerebral bleed*" or intracerebral or intracranial or stroke* or infection* or meningitis or subarachnoid or h#emorrhag*)<br>S12 S8 OR S9 OR S10 OR S11<br>S13 S7 AND S12<br>S14 S1 OR S2 OR S13<br>S15 (MH "Animals+")<br>S16 (MH "Human")<br>S17 S15 NOT (S15 AND S16)<br>S18 TI veterinary or rabbit or rabbits or animal or animals or mouse or mice or rodent or rodents or rat or rats or hamster* or pig or pigs or porcine or horse* or equine or cow or cows or bovine or goat or goats or sheep or ovine or canine or dog or dogs or feline or cat or cats or zebrafish<br>S19 S17 OR S18<br>S20 S14 NOT S19<br>S21 S14 NOT S19 [Limit to Commentary]<br>S22 S14 NOT S19 [Limit to Editorial]<br>S23 S21 OR S22<br>S24 S20 NOT S23
**Scopus**	(TITLE-ABS-KEY ("augmented renal clearance" OR "augmented kidney clearance") OR TITLE-ABS-KEY ((((increas* OR enhanc* OR high*) W/2 ("kidney

		function" OR "kidney clearance" OR "renal function" OR "renal clearance")) OR ((increas* OR high*) W/2 ("creatinine clearance" OR "drug clearance" OR "med* clearance")) OR (ultrafiltrat* W/3 (kidney OR renal)) OR "glomerular hyperfiltration") AND (icu OR "intensive care" OR "critical care" OR "critical* ill*" OR "acute care" OR sepsis OR "septic shock" OR trauma OR "brain injur*" OR "brain bleed*" OR "cerebral bleed*" OR intracerebral OR intracranial OR stroke* OR infection* OR meningiti s OR subarachnoid OR hemorrhag* OR haemorrhag*))) AND NOT TITLE (veterinary OR rabbit OR rabbits OR animal OR animals OR mouse OR mice OR rodent OR rodents OR rat OR rats OR hamster* OR pig OR pigs OR po rcine OR horse* OR equine OR cow OR cows OR bovine OR goat OR goats OR sheep OR ovine OR canine OR dog OR dogs OR feline OR cat OR cats OR zebrafish) AND (EXCLUDE (DOCTYPE , "ed"))
**Cochrane Library** via Wiley	#1	augmented renal clearance
	#2	augmented kidney clearance
	#3	(increas* or enhanc* or high*) NEAR/2 ("kidney function" or "kidney clearance" or "renal function" or "renal clearance")
	#4	(increas* or high*) NEAR/2 ("creatinine clearance" or "drug clearance" or "med* clearance")
	#5	(ultrafiltrat* NEAR/3 (kidney or renal))
	#6	"glomerular hyperfiltration"
	#7	{OR #3-#6}
	#8	[mh "intensive care units"[mj]]
	#9	ICU or "intensive care" or "critical care" or "critical* ill*" or "acute care"
	#10	[mh "critical care"[mj]]
	#11	sepsis or "septic shock" or trauma or "brain injur*" or "brain bleed*" or "cerebral bleed*" or intracerebral or intracranial or stroke* or infection* or meningitis or subarachnoid or hemorrhag* or haemorrhag*
	#12	{OR #8-#11}
	#13	#7 AND #12
	#14	#1 OR #2 OR #13
**ProQuest Dissertations and Theses Global**		noft("augmented renal clearance" OR "augmented kidney clearance") OR noft(((increas* NEAR/2 ("kidney function" OR "kidney clearance" OR "renal function" OR "renal clearance")) OR (enhanc* NEAR/2 ("kidney function" OR "kidney clearance" OR "renal function" OR "renal clearance")) OR (high* NEAR/2 ("kidney function" OR "kidney clearance" OR "renal function" OR "renal clearance")) OR (increas* NEAR/2 ("creatinine clearance" OR "drug clearance" OR "med* clearance")) OR (high* NEAR/2 ("creatinine clearance" OR "drug clearance" OR "med* clearance")) OR (ultrafiltrat* NEAR/3 (kidney OR renal)) OR "glomerular hyperfiltration") AND (icu OR "intensive care" OR "critical care" OR "critical* ill*" OR "acute care" OR sepsis OR "septic shock" OR trauma OR ("brain injured" OR "brain injuries" OR "brain injury") OR "brain bleed*" OR "cerebral bleed*" OR intracerebral OR intracranial OR stroke* OR infection*

	OR meningitis OR subarachnoid OR hemorrhag* OR haemorrhag*)) NOT ti(veterinary OR rabbit OR rabbits OR animal OR animals OR mouse OR mice OR rodent OR rodents OR rat OR rats OR hamster* OR pig OR pigs OR porc ine OR horse* OR equine OR cow OR cows OR bovine OR goat OR goats OR sheep OR ovine OR canine OR dog OR dogs OR feline OR cat OR cats OR zebrafish)
**Google Scholar**	augmented renal clearance OR enhanced renal function OR enhanced renal clearance OR increased kidney function OR increased kidney clearance

Table S2. Appraisal of individual studies included in this review.

Prevalence/Incidence Studies	A	B	C	D	E	F	G	H	I	Total
Adnan (2014) [1]	Yes	Yes	No	Yes	Yes	Yes	Yes	Yes	Yes	**8/9**
Abdel el Naeem (2017) [2]	Yes	Unclear	No	Yes	Yes	Yes	Yes	Yes	Yes	**7/9**
Aréchiga-Alvarado et al. [3]	Yes	Yes	Yes	Yes	Yes	Yes	Yes	Yes	Yes	**9/9**
Aitullina (2019) [4]	Yes	Yes	No	Yes	Yes	No	Yes	Yes	Yes	**7/9**
Baptista (2011) [5]	Yes	Yes	No	Yes	Yes	Yes	Yes	Yes	Yes	**8/9**
Baptista (2012) [6]	Yes	Yes	No	Yes	Yes	Yes	Yes	Yes	Yes	**8/9**
Baptista (2014) [7]	Yes	Yes	Yes	Yes	Yes	Yes	Yes	Yes	Yes	**9/9**
Baptista et al.(2014) [8]	Yes	Yes	Yes	Yes	Yes	Yes	Yes	Yes	Yes	**9/9**
Baptista (2020) [9]	Yes	Yes	Yes	Yes	Yes	Yes	Yes	Yes	Yes	**9/9**
Barletta (2016) [10]	Yes	Yes	No	Yes	Yes	Yes	Yes	Yes	Yes	**8/9**
Barletta (2017) [11]	Yes	Yes	No	Yes	Yes	Yes	Yes	Yes	Yes	**8/9**
Barrasa (2020) [12]	Yes	Yes	No	Yes	Yes	Yes	Yes	Yes	Yes	**8/9**
Bricheux (2019) [13]	Unclear	Yes	Yes	Yes	Yes	No	Yes	Yes	Yes	**7/9**
Brown (2020) [14]	Yes	Yes	No	Yes	No	Yes	Yes	Yes	Yes	**7/9**
Burnham (2017) [15]	Yes	Yes	Yes	Yes	Yes	No	Yes	Yes	Yes	**8/9**
Campassi (2014) [16]	Yes	Yes	No	Yes	Yes	Yes	Yes	No	Yes	**7/9**
Carlier (2013) [17]	Yes	Yes	No	Yes	Yes	Yes	Yes	Yes	Yes	**8/9**
Carrie (2018a) [18]	Yes	Yes	No	Yes	Yes	Yes	Yes	Yes	Yes	**8/9**
Carrie (2018b) [19]	Yes	Yes	No	Yes	Yes	Yes	Yes	Yes	Yes	**8/9**
Carrie (2019a) [20]	Yes	Yes	No	Yes	Yes	Yes	Yes	Yes	Yes	**8/9**
Carrie (2019b) [21]	No	Yes	No	No	Yes	Yes	Yes	Yes	Yes	**6/9**
Carrie (2020) [22]	Unclear	Yes	No	Yes	Yes	No	Yes	Yes	Yes	**6/9**
Chen (2020) [23]	Yes	Yes	No	Yes	Yes	No	Yes	Yes	Yes	**7/9**
Chu (2016) [24]	Yes	Unclear	No	Yes	Yes	No	Yes	Yes	Yes	**6/9**
Chu (2019) [25]	Yes	Yes	Yes	Yes	Yes	No	Yes	Yes	Yes	**8/9**
Claus (2013) [26]	Yes	Yes	No	Yes	Yes	Yes	Yes	Yes	Yes	**8/9**
Cojutti (2020) [27]	No	Yes	No	Yes	Yes	No	Yes	Yes	Yes	**6/9**

Dhaese et al. (2018) [28]	Yes	Yes	Yes	Yes	Yes	Yes	Yes	Yes	Yes	**9/9**
Declercq (2016) [29]	Yes	Yes	Yes	Yes	Yes	Yes	Yes	Yes	Yes	**9/9**
DeWaele (2015) [30]	Yes	Yes	Yes	Yes	Yes	Yes	Yes	Yes	Yes	**9/9**
Dias (2015) [31]	Yes	Yes	No	Yes	Yes	No	Yes	Yes	Yes	**7/9**
Ehmann (2017) [32]	No	Yes	No	Yes	Yes	No	Yes	Yes	Yes	**6/9**
Eidelson et al.[33]	No	Yes	No	Yes	Yes	Yes	Yes	Yes	Yes	**7/9**
Fuster-Lluch (2008) [34]	Yes	Yes	Yes	Yes	Yes	Yes	Yes	Yes	Yes	**9/9**
Gijsen (2020) [35]	Yes	Yes	Yes	Yes	Yes	Yes	Yes	Yes	Yes	**9/9**
Grootaert (2012) [36]	Yes	Yes	Yes	Yes	Yes	Yes	Yes	Yes	Yes	**9/9**
Helset (2020) [37]	Yes	Yes	No	Yes	Yes	Yes	Yes	Yes	Yes	**8/9**
Hirai (2016) [38]	Yes	Yes	Yes	Yes	Yes	No	Yes	Yes	Yes	**8/9**
Huttner (2015) [39]	Yes	Unclear	No	Yes	Yes	No	Yes	Yes	Yes	**6/9**
Ishii (2018) [40]	Yes	Yes	No	Yes	No	No	Yes	No	Yes	**5/9**
Izumisawa (2019) [41]	Unclear	Yes	Yes	Yes	Yes	No	Yes	Yes	Yes	**7/9**
Joynt (2001) [42]	Yes	Yes	No	Yes	Yes	Yes	Yes	No	Yes	**7/9**
Kawano et al.(2016) [43]	Yes	Yes	Yes	Yes	Yes	Yes	Yes	Yes	Yes	**9/9**
Kawano (2018) [44]	Yes	Yes	Yes	Yes	Yes	Yes	Yes	Yes	Yes	**9/9**
Lannou (2020) [45]	Yes	Yes	No	Yes	Yes	Yes	Yes	Yes	Yes	**8/9**
Lannou editorial letter (2020) [46]	Yes	Yes	No	Yes	Yes	Yes	Yes	Yes	Yes	**8/9**
Lautrette (2012) [47]	Yes	Yes	No	Yes	Yes	Yes	Yes	Yes	Yes	**8/9**
May (2015) [48]	Yes	Yes	No	Yes	Yes	Yes	Yes	Yes	Yes	**8/9**
Minkute (2013) [49]	Unclear	Yes	No	Yes	Yes	No	Yes	Yes	Yes	**6/9**
Minville (2011) [50]	Yes	Yes	Yes	Yes	Yes	Yes	Yes	No	Yes	**8/9**
Morbitzer (2019) [51]	Yes	Yes	Yes	Yes	Yes	Yes	Yes	Yes	Yes	**9/9**
Mulder (2019) [52]	Yes	Yes	No	Yes	Yes	Yes	Yes	Yes	Yes	**8/9**
Nei (2020) [53]	Yes	Yes	Yes	Yes	Yes	No	Yes	Yes	Yes	**8/9**
Ollivier (2019) [54]	Yes	Yes	No	Yes	Yes	Yes	Yes	Yes	Yes	**8/9**
Ramos (2017)[55]	Yes	Yes	No	No	Unclear	Yes	Yes	Yes	Yes	**6/9**
Ruiz (2015) [56]	Yes	Yes	Yes	Yes	Yes	Yes	Yes	Yes	Yes	**9/9**
Saito (2020) [57]	Yes	Yes	No	Yes	Yes	No	Yes	Yes	Yes	**7/9**
Saour (2016) [58]	Yes	Yes	Yes	Yes	Yes	No	Yes	Yes	Yes	**8/9**
Steinke (2015) [59]	Yes	Yes	No	Yes	Unclear	Yes	No	Yes	Yes	**6/9**

Tamatsukuri (2018) [60]	Yes	Yes	No	Yes	Yes	No	Yes	Yes	Yes	**7/9**
Tsai (2018) [61]	Yes	Yes	No	Yes	Yes	Yes	Yes	Yes	Yes	**8/9**
Udy (2013) [62]	Yes	Yes	No	Yes	Yes	Yes	Yes	Yes	Yes	**8/9**
Udy (2013b) [63]	Yes	Yes	No	Yes	Yes	Yes	Yes	Yes	Yes	**8/9**
Udy (2014) [64]	Yes	Yes	Yes	Yes	Yes	Yes	Yes	Yes	Yes	**9/9**
Udy (2017) [65]	Yes	Yes	No	Yes	Yes	Yes	Yes	Yes	Yes	**8/9**
Udy (2018) [66]	No	Yes	Yes	Yes	Yes	Yes	Yes	Yes	Yes	**8/9**
Villaneuva (2019) [67]	Yes	Yes	No	Yes	Yes	No	Yes	Yes	Yes	**7/9**
Weber (2019) [68]	No	Yes	No	Yes	Yes	Yes	Yes	No	Yes	**6/9**
Wong (2018) [69]	Unclear	Yes	Yes	Yes	No	No	Yes	Yes	Yes	**6/9**
Wu (2019) [70]	Yes	Yes	No	Yes	Yes	Yes	Yes	Yes	Yes	**8/9**

A: Was the sample frame appropriate to address the target population? **B:** Were study participants sampled in an appropriate way? C: Was the sample size adequate? **D:** Were the study subjects and the setting described in detail? **E:** Was the data analysis conducted with sufficient coverage of the identified sample? **F:** Were valid methods used for the identification of the condition? **G:** Was the condition measured in a standard, reliable way for all participants? **H:** Was there appropriate statistical analysis? **I:** Was the response rate adequate, and if not, was the low response rate managed appropriately?

References:

1. Adnan S, Ratnam S, Kumar S, Paterson D, Lipman J, Roberts J, Udy AA, (2014) Select critically ill patients at risk of augmented renal clearance: experience in a Malaysian intensive care unit. Anaesthesia and Intensive Care 42: 715-722
2. Abdel El Naeem HEM, Abdelhamid MHE, Atteya DAM, (2017) Impact of augmented renal clearance on enoxaparin therapy in critically ill patients. Egyptian Journal of Anaesthesia 33: 113-117
3. Arechiga-Alvarado NA, Medellin-Garibay SE, Milan-Segovia RDC, Ortiz-Alvarez A, Magana-Aquino M, Romano-Moreno S, (2020) Population Pharmacokinetics of Amikacin Administered Once Daily in Patients with Different Renal Functions. Antimicrobial agents and chemotherapy 64
4. Aitullina A, Krumina A, Purvina S, (2019) Augmented clearance in patients with colistin therapy in intensive care units. International Journal of Clinical Pharmacy 41: 310
5. Baptista JP, Udy AA, Sousa E, Pimentel J, Wang L, Roberts JA, Lipman J, (2011) A comparison of estimates of glomerular filtration in critically ill patients with augmented renal clearance. Critical care (London, England) 15: R139
6. Baptista JP, Sousa E, Martins PJ, Pimentel JM, (2012) Augmented renal clearance in septic patients and implications for vancomycin optimisation. International journal of antimicrobial agents 39: 420-423
7. Baptista JP, Roberts JA, Sousa E, Freitas R, Deveza N, Pimentel J, (2014) Decreasing the time to achieve therapeutic vancomycin concentrations in critically ill patients: developing and testing of a dosing nomogram. Critical care (London, England) 18: 654
8. Baptista JP, Neves M, Rodrigues L, Teixeira L, Pinho J, Pimentel J, (2014) Accuracy of the estimation of glomerular filtration rate within a population of critically ill patients. Journal of nephrology 27: 403-410
9. Baptista JP, Martins PJ, Marques M, Pimentel JM, (2020) Prevalence and Risk Factors for Augmented Renal Clearance in a Population of Critically Ill Patients. Journal of intensive care medicine 35: 1044-1052
10. Barletta JF, Mangram AJ, Byrne M, Hollingworth AK, Sucher JF, Ali-Osman FR, Shirah GR, Dzandu JK, (2016) The importance of empiric antibiotic dosing in critically ill trauma patients: Are we under-dosing based on augmented renal clearance and inaccurate renal clearance estimates? The journal of trauma and acute care surgery 81: 1115-1121
11. Barletta JF, Mangram AJ, Byrne M, Sucher JF, Hollingworth AK, Ali-Osman FR, Shirah GR, Haley M, Dzandu JK, (2017) Identifying augmented renal clearance in trauma patients: Validation of the Augmented Renal Clearance in Trauma Intensive Care scoring system. The journal of trauma and acute care surgery 82: 665-671
12. Barrasa H, Soraluce A, Uson E, Sainz J, Martin A, Sanchez-Izquierdo JA, Maynar J, Rodriguez-Gascon A, Isla A, (2020) Impact of augmented renal clearance on the pharmacokinetics of linezolid: Advantages of continuous infusion from a pharmacokinetic/pharmacodynamic perspective. International journal of infectious diseases : IJID : official publication of the International Society for Infectious Diseases 93: 329-338
13. Bricheux A, Lenggenhager L, Hughes S, Karmime A, Lescuyer P, Huttner A, (2019) Therapeutic drug monitoring of imipenem and the incidence of toxicity and failure in hospitalized patients: a retrospective cohort study. Clinical microbiology and infection : the official publication of the European Society of Clinical Microbiology and Infectious Diseases 25: 383.e381-383.e384
14. Brown A, Lavelle R, Gerlach A, (2020) Discordance of renal drug dosing using estimated creatinine clearance and measured urine creatinine clearance in hospitalized adults: A retrospective cohort study. International Journal of Critical Illness and Injury Science 10: S1-S5
15. Burnham JP, Micek ST, Kollef MH, (2017) Augmented renal clearance is not a risk factor for mortality in Enterobacteriaceae bloodstream infections treated with appropriate empiric antimicrobials. PLoS ONE 12: e0180247
16. Campassi ML, Gonzalez MC, Masevicius FD, Vazquez AR, Moseinco M, Navarro NC, Previgliano L, Rubatto NP, Benites MH, Estenssoro E, Dubin A, (2014) Augmented renal clearance in critically ill patients: incidence, associated factors and

effects on vancomycin treatment. Incremento da depuracao renal em pacientes gravemente enfermos: incidencia, fatores associados e efeitos no tratamento com vancomicina 26: 13-20

17. Carlier M, Carrette S, Roberts JA, Stove V, Verstraete A, Hoste E, Depuydt P, Decruyenaere J, Lipman J, Wallis SC, De Waele JJ, (2013) Meropenem and piperacillin/tazobactam prescribing in critically ill patients: does augmented renal clearance affect pharmacokinetic/pharmacodynamic target attainment when extended infusions are used? Critical care (London, England) 17: R84

18. Carrie C, Lannou A, Rubin S, De Courson H, Petit L, Biais M, (2019) Augmented renal clearance in critically ill trauma patients: A pathophysiologic approach using renal vascular index. Anaesthesia, critical care & pain medicine 38: 371-375

19. Carrie C, Petit L, d'Houdain N, Sauvage N, Cottenceau V, Lafitte M, Foumenteze C, Hisz Q, Menu D, Legeron R, Breilh D, Sztark F, (2018) Association between augmented renal clearance, antibiotic exposure and clinical outcome in critically ill septic patients receiving high doses of beta-lactams administered by continuous infusion: a prospective observational study. International journal of antimicrobial agents 51: 443-449

20. Carrie C, Legeron R, Petit L, Ollivier J, Cottenceau V, d'Houdain N, Boyer P, Lafitte M, Xuereb F, Sztark F, Breilh D, Biais M, (2018) Higher than standard dosing regimen are needed to achieve optimal antibiotic exposure in critically ill patients with augmented renal clearance receiving piperacillin-tazobactam administered by continuous infusion. Journal of critical care 48: 66-71

21. Carrie C, Bentejac M, Cottenceau V, Masson F, Petit L, Cochard JF, Sztark F, (2018) Association between augmented renal clearance and clinical failure of antibiotic treatment in brain-injured patients with ventilator-acquired pneumonia: A preliminary study. Anaesthesia, critical care & pain medicine 37: 35-41

22. Carrie C, Delzor F, Roure S, Dubuisson V, Petit L, Molimard M, Breilh D, Biais M, (2020) Population Pharmacokinetic Study of the Suitability of Standard Dosing Regimens of Amikacin in Critically Ill Patients with Open-Abdomen and Negative-Pressure Wound Therapy. Antimicrobial agents and chemotherapy 64

23. Chen Y, Liu L, Zhu M, (2020) Effect of augmented renal clearance on the therapeutic drug monitoring of vancomycin in patients after neurosurgery. The Journal of international medical research 48: 300060520949076

24. Chu, Y.; Luo, Y.; Qu, L.; Zhao, C.; Jiang, M. Application of vancomycin in patients with varying renal function, especially those with augmented renal clearance. Pharmaceut. Biol. 2016, 54, 2802–2806.

25. Chu Y, Luo Y, Jiang M, Zhou B, (2020) Application of vancomycin in patients with augmented renal clearance. European journal of hospital pharmacy : science and practice 27: 276-279

26. Claus BOM, Hoste EA, Colpaert K, Robays H, Decruyenaere J, De Waele JJ, (2013) Augmented renal clearance is a common finding with worse clinical outcome in critically ill patients receiving antimicrobial therapy. Journal of critical care 28: 695-700

27. Cojutti PG, Lazzarotto D, Candoni A, Dubbini MV, Zannier ME, Fanin R, Pea F, (2020) Real-time TDM-based optimization of continuous-infusion meropenem for improving treatment outcome of febrile neutropenia in oncohaematological patients: results from a prospective, monocentric, interventional study. The Journal of antimicrobial chemotherapy 75: 3029-3037

28. Dhaese SAM, Roberts JA, Carlier M, Verstraete AG, Stove V, De Waele JJ, (2018) Population pharmacokinetics of continuous infusion of piperacillin in critically ill patients. International journal of antimicrobial agents 51: 594-600

29. Declercq P, Nijs S, D'Hoore A, Van Wijngaerden E, Wolthuis A, de Buck van Overstraeten A, Wauters J, Spriet I, (2016) Augmented renal clearance in non-critically ill abdominal and trauma surgery patients is an underestimated phenomenon: A point prevalence study. The journal of trauma and acute care surgery 81: 468-477

30. De Waele JJ, Dumoulin A, Janssen A, Hoste EA, (2015) Epidemiology of augmented renal clearance in mixed ICU patients. Minerva anestesiologica 81: 1079-1085

31. Dias C, Gaio AR, Monteiro E, Barbosa S, Cerejo A, Donnelly J, Felgueiras O, Smielewski P, Paiva JA, Czosnyka M, (2015) Kidney-Brain Link in Traumatic Brain Injury Patients? A preliminary report. Neurocritical Care 22: 192-201

32. Ehmann L, Zoller M, Minichmayr IK, Scharf C, Maier B, Schmitt MV, Hartung N, Huisinga W, Vogeser M, Frey L, Zander J, Kloft C, (2017) Role of renal function in risk assessment of target non-attainment after standard dosing of meropenem in critically ill patients: a prospective observational study. Critical Care 21: 1-14
33. Eidelson SA, Mulder MB, Rattan R, Karcutskie CA, Meizoso JP, Madiraju SK, Lineen EB, Schulman CI, Namias N, (2018) Incidence and Functional Significance of Augmented Renal Clearance in Trauma Patients at High Risk for Venous Thromboembolism. Journal of the American College of Surgeons 227: S80-S81
34. Fuster-Lluch O, Geronimo-Pardo M, Peyro-Garcia R, Lizan-Garcia M, (2008) Glomerular hyperfiltration and albuminuria in critically ill patients. Anaesthesia and intensive care 36: 674-680
35. Gijsen M, Huang CY, Flechet M, Van Daele R, Declercq P, Debaveye Y, Meersseman P, Meyfroidt G, Wauters J, Spriet I, (2020) Development and External Validation of an Online Clinical Prediction Model for Augmented Renal Clearance in Adult Mixed Critically Ill Patients: The Augmented Renal Clearance Predictor. Critical care medicine
36. Grootaert V, Spriet I, Decoutere L, Debaveye Y, Meyfroidt G, Willems L, (2012) Augmented renal clearance in the critically ill: Fiction or fact? International Journal of Clinical Pharmacy 34: 143
37. Helset E, Nordøy I, Sporsem H, Bakke VD, Bugge JF, Gammelsrud KW, Zucknick M, Lippe E, von der Lippe E, (2020) Factors increasing the risk of inappropriate vancomycin therapy in ICU patients: A prospective observational study. Acta Anaesthesiologica Scandinavica 64: 1295-1304
38. Hirai K, Ishii H, Shimoshikiryo T, Shimomura T, Tsuji D, Inoue K, Kadoiri T, Itoh K, (2016) Augmented renal clearance in patients with febrile neutropenia is associated with increased risk for subtherapeutic concentrations of vancomycin. Therapeutic drug monitoring 38: 706-710
39. Huttner A, Von Dach E, Renzoni A, Huttner BD, Affaticati M, Pagani L, Daali Y, Pugin J, Karmime A, Fathi M, Lew D, Harbarth S, (2015) Augmented renal clearance, low beta-lactam concentrations and clinical outcomes in the critically ill: an observational prospective cohort study. International journal of antimicrobial agents 45: 385-392
40. Ishii H, Hirai K, Sugiyama K, Nakatani E, Kimura M, Itoh K, (2018) Validation of a Nomogram for Achieving Target Trough Concentration of Vancomycin: Accuracy in Patients With Augmented Renal Function. Therapeutic drug monitoring 40: 693-698
41. Izumisawa T, Kaneko T, Soma M, Imai M, Wakui N, Hasegawa H, Horino T, Takahashi N, (2019) Augmented Renal Clearance of Vancomycin in Hematologic Malignancy Patients. Biological & pharmaceutical bulletin 42: 2089-2094
42. Joynt GM, Lipman J, Gomersall CD, Young RJ, Wong EL, Gin T, (2001) The pharmacokinetics of once-daily dosing of ceftriaxone in critically ill patients. The Journal of antimicrobial chemotherapy 47: 421-429
43. Kawano Y, Morimoto S, Izutani Y, Muranishi K, Kaneyama H, Hoshino K, Nishida T, Ishikura H, (2016) Augmented renal clearance in Japanese intensive care unit patients: a prospective study. Journal of intensive care 4: 62
44. Kawano Y, Maruyama J, Hokama R, Koie M, Nagashima R, Hoshino K, Muranishi K, Nakashio M, Nishida T, Ishikura H, (2018) Outcomes in patients with infections and augmented renal clearance: A multicenter retrospective study. PLoS ONE 13: e0208742
45. Lannou A, Carrie C, Rubin S, Cane G, Cottenceau V, Petit L, Biais M, (2020) Salt wasting syndrome in brain trauma patients: a pathophysiologic approach using sodium balance and urinary biochemical analysis. BMC neurology 20: 190
46. Lannou A, Carrie C, Rubin S, De Courson H, Biais M, (2020) Renal response after traumatic brain injury: A pathophysiological relationship between augmented renal clearance and salt wasting syndrome? Anaesthesia, critical care & pain medicine 39: 239-241
47. Lautrette A, Phan TN, Ouchchane L, Aithssain A, Tixier V, Heng AE, Souweine B, (2012) High creatinine clearance in critically ill patients with community-acquired acute infectious meningitis. BMC nephrology 13: 124
48. May CC, Arora S, Parli SE, Fraser JF, Bastin MT, Cook AM, (2015) Augmented Renal Clearance in Patients with Subarachnoid Hemorrhage. Neurocritical care 23: 374-379

49. Minkute R, Briedis V, Steponaviciute R, Vitkauskiene A, Maciulaitis R, (2013) Augmented renal clearance--an evolving risk factor to consider during the treatment with vancomycin. Journal of clinical pharmacy and therapeutics 38: 462-467
50. Minville V, Asehnoune K, Ruiz S, Breden A, Georges B, Seguin T, Tack I, Jaafar A, Saivin S, Fourcade O, Samii K, Conil JM, (2011) Increased creatinine clearance in polytrauma patients with normal serum creatinine: a retrospective observational study. Critical care (London, England) 15: R49
51. Morbitzer KA, Rhoney DH, Dehne KA, Jordan JD, (2019) Enhanced renal clearance and impact on vancomycin pharmacokinetic parameters in patients with hemorrhagic stroke. Journal of intensive care 7: 51
52. Mulder MB, Eidelson SA, Sussman MS, Schulman CI, Lineen EB, Iyenger RS, Namias N, Proctor KG, (2019) Risk Factors and Clinical Outcomes Associated With Augmented Renal Clearance in Trauma Patients. The Journal of surgical research 244: 477-483
53. Nei AM, Kashani KB, Dierkhising R, Barreto EF, (2020) Predictors of Augmented Renal Clearance in a Heterogeneous ICU Population as Defined by Creatinine and Cystatin C. Nephron 144: 313-320
54. Ollivier J, Carrie C, d'Houdain N, Djabarouti S, Petit L, Xuereb F, Legeron R, Biais M, Breilh D, (2019) Are Standard Dosing Regimens of Ceftriaxone Adapted for Critically Ill Patients with Augmented Creatinine Clearance? Antimicrobial agents and chemotherapy 63
55. Ramos A, Acharta F, Perezlindo M, Lovesio L, Gauna Antonelli P, Dogliotti A, Lovesio C, (2017) Factors that predict supranormal glomerular filtration in critical diseases. Critical Care 21
56. Ruiz S, Minville V, Asehnoune K, Virtos M, Georges B, Fourcade O, Conil JM, (2015) Screening of patients with augmented renal clearance in ICU: taking into account the CKD-EPI equation, the age, and the cause of admission. Annals of intensive care 5: 49
57. Saito K, Kamio S, Ito K, Suzuki N, Abe K, Goto T, (2020) A simple scoring method to predict augmented renal clearance in haematologic malignancies. Journal of clinical pharmacy and therapeutics
58. Saour M, Klouche K, Deras P, Damou A, Capdevila X, Charbit J, (2016) Assessment of modification of diet in renal disease equation to predict reference serum creatinine value in severe trauma patients: Lessons from an obser vational study of 775 cases. Annals of Surgery 263: 814-820
59. Steinke T, Moritz S, Beck S, Gnewuch C, Kees MG, (2015) Estimation of creatinine clearance using plasma creatinine or cystatin C: a secondary analysis of two pharmacokinetic studies in surgical ICU patients. BMC anesthesiology 15: 62
60. Tamatsukuri T, Ohbayashi M, Kohyama N, Kobayashi Y, Yamamoto T, Fukuda K, Nakamura S, Miyake Y, Dohi K, Kogo M, (2018) The exploration of population pharmacokinetic model for meropenem in augmented renal clearance and investigation of optimum setting of dose. Journal of infection and chemotherapy : official journal of the Japan Society of Chemotherapy 24: 834-840
61. Tsai D, Udy AA, Stewart PC, Gourley S, Morick NM, Lipman J, Roberts JA, (2018) Prevalence of augmented renal clearance and performance of glomerular filtration estimates in Indigenous Australian patients requiring intensive care admission. Anaesthesia and intensive care 46: 42-50
62. Udy AA, Roberts JA, Shorr AF, Boots RJ, Lipman J, (2013) Augmented renal clearance in septic and traumatized patients with normal plasma creatinine concentrations: identifying at-risk patients. Critical care (London, England) 17: R35
63. Udy AA, Morton FJA, Nguyen-Pham S, Jarrett P, Lassig-Smith M, Stuart J, Dunlop R, Starr T, Boots RJ, Lipman J, (2013) A comparison of CKD-EPI estimated glomerular filtration rate and measured creatinine clearance in recently admitted critically ill patients with normal plasma creatinine concentrations. BMC nephrology 14: 250
64. Udy AA, Baptista JP, Lim NL, Joynt GM, Jarrett P, Wockner L, Boots RJ, Lipman J, (2014) Augmented Renal Clearance in the ICU: Results of a Multicenter Observational Study of Renal Function in Critically Ill Patients With Normal Plasma Creatinine Concentrations. Crit Care Med 42: 520-527

65. Udy AA, Jarrett P, Lassig-Smith M, Stuart J, Starr T, Dunlop R, Deans R, Roberts JA, Senthuran S, Boots R, Bisht K, Bulmer AC, Lipman J, (2017) Augmented Renal Clearance in Traumatic Brain Injury: A Single-Center Observational Study of Atrial Natriuretic Peptide, Cardiac Output, and Creatinine Clearance. Journal of neurotrauma 34: 137-144

66. Udy AA, Dulhunty JM, Roberts JA, Davis JS, Webb SAR, Bellomo R, Gomersall C, Shirwadkar C, Eastwood GM, Myburgh J, Paterson DL, Starr T, Paul SK, Lipman J, Investigators BI, Group ACT, (2017) Association between augmented renal clearance and clinical outcomes in patients receiving beta-lactam antibiotic therapy by continuous or intermittent infusion: a nested cohort study of the BLING-II randomised, placebo-controlled, clinical trial. International journal of antimicrobial agents 49: 624-630

67. Villanueva RD, Talledo O, Neely S, White B, Celii A, Cross A, Kennedy R, (2019) Vancomycin dosing in critically ill trauma patients: The VANCTIC Study. The journal of trauma and acute care surgery 87: 1164-1171

68. Weber N, Jackson K, McWhinney B, Ungerer J, Kennedy G, Lipman J, Roberts JA, (2019) Evaluation of pharmacokinetic/pharmacodynamic and clinical outcomes with 6-hourly empiric piperacillin-tazobactam dosing in hematological malignancy patients with febrile neutropenia. Journal of infection and chemotherapy : official journal of the Japan Society of Chemotherapy 25: 503-508

69. Wong G, Briscoe S, McWhinney B, Ally M, Ungerer J, Lipman J, Roberts JA, (2018) Therapeutic drug monitoring of beta-lactam antibiotics in the critically ill: direct measurement of unbound drug concentrations to achieve appropriate drug exposures. The Journal of antimicrobial chemotherapy 73: 3087-3094

70. Wu CC, Tai CH, Liao WY, Wang CC, Kuo CH, Lin SW, Ku SC, (2019) Augmented renal clearance is associated with inadequate antibiotic pharmacokinetic/pharmacodynamic target in Asian ICU population: a prospective observational study. Infection and drug resistance 12: 2531-2541
